# Purifying Tidal Rivers

**Published:** 1921-09-24

**Authors:** 


### Purifying Tidal Rivers.

The drought of the late summer has turned the thoughts of sanitary authorities to the problem of increasing our water supply. The water for our large towns is brought for the most part from far-off mountain springs and lakes, where it is comparatively pure, while we dare not drink from the rivers that flow through our cities. Yet in America it has been proved practicable to make the water of even tidal rivers potable.

This treatment of river water is part of a great experiment of the United States Public Health Service. This department has taken over a Government reservation at Perryville, Maryland, and has built there a model village which derives its drinking water from the river Susquehanna. This water, says the report of the Health Service Bureau, "is naturally in bad condition, being contaminated in several highly variable ways." In face of this rather alarming statement it is interesting to learn how it is made fit for human consumption. The raw water is pumped through 30-inch mains into settling tanks. Samples of the water entering the mains is taken every two hours, because the amount of turbidity in it varies much. Sometimes it is ten parts to a million, sometimes one hundred, and according to the degree of turbidity is the amount of aluminium sulphate which is added as a precipitate and coagulant, the allowance being 0.6 grain of the alum for every added ten parts of turbidity. The alkalinity of the water also varies during the day, and when this drops below fourteen parts per million soda ash is added. These chemicals are added to the water in the settling tanks into which the mains deliver it, and where it is allowed to remain for two hours. On release from the tanks, the water flows through rapid sand filters, passing through three feet of sand and eight inches of stone and gravel at the rate of two inches in fifty-five seconds. This, however, does not end the treatment, for a bacteriological examination is made of the water, which is then treated with liquid chlorine in proportion to the bacteria found in it. The raw water has sometimes been found to contain as many as 7,860 bacteria per cubic centimeter, and the average is 2,630, whereas after treatment the number is reduced to less than one.

